# Electromyography signal based hand gesture classification system using Hilbert Huang transform and deep neural networks

**DOI:** 10.1016/j.heliyon.2024.e32211

**Published:** 2024-05-31

**Authors:** Mary Vasanthi S, Haiter Lenin A, Yasser Fouad, Manzoore Elahi M. Soudagar

**Affiliations:** a Department of Electronics and Communication Engineering, St Xavier's Catholic College of Engineering, Nagercoil, Tamilnadu, India; b School of Mechanical and Chemical Engineering, WOLLO University, Kombolcha Institute of Technology, Kombolcha, Post Box No: 208, Ethiopia; c Department of Applied Mechanical Engineering, College of Applied Engineering, Muzahimiyah Branch, King Saud University, Riyadh, Saudi Arabia; d Faculty of Engineering, Lishui University, 323000, Lishui, Zhejiang, People's Republic of China

**Keywords:** Electromyography, Hilbert huang transform, Wavelet transform, Deep neural network, Learning

## Abstract

This research aims to provide the groundwork for smartly categorizing hand movements for use with prosthetic hands. The hand motions are classified using surface electromyography (sEMG) data. In reaction to a predetermined sequence of fibre activation, every single one of our muscles contracts. They could be useful in developing control protocols for bio-control systems, such human-computer interaction and upper limb prostheses. When focusing on hand gestures, data gloves and vision-based approaches are often used. The data glove technique requires tedious and unnatural user engagement, whereas the vision-based solution requires significantly more expensive sensors. This research offered a Deep Neural Network (DNN) automated hand gesticulation recognition system based on electromyography to circumvent these restrictions. This work primarily aims to augment the concert of the hand gesture recognition system via the use of an artificial classifier. To advance the recognition system's classification accuracy, this study explains how to build models of neural networks and how to use signal processing methods. By locating the Hilbert Huang Transform (HHT), one may get the essential properties of the signal. When training a DNN classifier, these characteristics are sent into it. The investigational results reveal that the suggested technique accomplishes a better categorization rate (98.5 % vs. the alternatives).

## 1 Introduction

The widespread use of electromyography (EMG) as a signal for the detection of various postures, gestures, and activities has led to its widespread adoption [1]. An electrical muscle potential (EMG) signal was produced by stimulating the neuronal circuits in the muscles [2]. Gathering data from muscles via their surface EMG signal is possible using surface electromyography (sEMG), a non-invasive approach [3]. A key component in human-computer interaction (HCI) systems, electrodes may record electrical activity. Input devices (such as motor control interfaces and armbands), assistive devices (such as wheelchairs, myoelectric powered prostheses, and assistive robots), and rehabilitation are just a few examples of the many fields that make use of these systems [4]. The conveyance of messages and information was facilitated by the use of hand gestures [5]. Hand gestures are used in Game play and regulating landing and takeoff processes [6]. They are crucial for the transfer of data between computers and people and were also used for communication reasons [7]. The use of one's muscles to control a prosthetic hand is a natural interface for sensing hand movements [8]. Assistive technology that can decipher surface electromyography (sEMG)-based hand motions is vital. Myoelectric control of prosthetic limbs is made possible by surface EMG signals [9]. Using a signal captured at the skin surface of the forearm, the motions of a healthy subject's hand and fingers were recognised [10]. Compared to electroencephalography (EEG) electrodes inserted in the skull, electromyography (EMG) gloves will provide more comfort to a hand amputee [11]. Because of this, EMG has more applications in biological, therapeutic, and HMI contexts [12,13]. An issue arises when surface electrode EMG signals are susceptible to distortion due to factors such as skin resistance and other electromagnetic fields [14]. This necessitates cautious processing and classification of EMG data in order to get trustworthy findings about myoelectric control [15]. This suggests that performance may be improved with careful feature set and classifier selection [16]. In this study, methods for denoising signals are presented [17] employing wavelets to reduce noise.

A variety of attributes are retrieved according to the application. The following factors were considered: finger alignment, palm position, thumb status, grip, and finger motions [18].

Time domain characteristics may not always provide more accurate results, which is their major downside. A feature set based on wavelets might be one possible option. With the use of characteristics derived from Wavelet Transform (WT), six finger motions may be classified using four EMG channels [19]. A mother wavelet must be specified in advance in order to find WT [20]. Using the incorrect mother wavelets might also lead to bad outcomes. It is possible that the Hilbert-Huang Transform (HHT) holds the solution to these issues. When processing data, the adaptive HHT may be used to deal with signals that do not adhere to a normal distribution. In HHT, the number of Intrinsic Mode Functions (IMFs), the basic building blocks of a signal, is kept modest and constant. Because of its adaptability, this time-domain decomposition approach is very successful. This decomposition is helpful for non-stationary signal analysis as it relies on the properties of the time-scale data. The HHT approach has a stellar reputation for feature extraction due to its dependability, adaptability, and longevity.

Researchers looked at EMG signal analysis using EMD (Empirical Mode Decomposition) [21]. This instance included computing the IMFs with the help of the time series signal [22]. Reducing the idea of mode mixing and removing noise is one technique to aid the IMFs [23]. Several IMFs may be acquired utilising EMD techniques [24]. Features that can depict motions and maintain their form when hand gestures are rotated or flipped are essential for dependable pattern recognition [25].

A variety of classifiers are also used in the process of hand movement recognition. SVMs and NNs are two well-established and successful classifiers [26]. Nevertheless, Deep Learning (DL) models have recently converged on a thrilling machine learning trend. Unlike narrow structures like SVM and K-Nearest Neighbour (KNN), deep architecture may depict intricate interactions well without needing a significant number of nodes. Data mining, picture processing and signal analysis are just a few of the many disciplines that benefit from these approaches [27].

A more modern and enhanced network that operates in shift and translation invariances is the Convolutional Neural Network (CNN) [28]. This image identification technique primarily targeted medical imaging images, such as those from MRI, CT, and fundus photography [29]. Using convolutional neural networks (CNNs) is one approach to improving the hand gesture detection system [30]. Theconvolution and the processing time of CNN were reduced [31].

Using a Deep Learning (DL) technique, the research automatically recognises and evaluates hand motions using EMG data, which is a significant advancement. This technique proposes training a DNN classifier to detect and classify hand movements using a set of characteristics collected from EMG data via HHT determination. Here is the rest of the paper's outline: Full details of the suggested approach are provided in Section 2. The Hilbert transform is demarcated in full in Section 3. In Section 4, we discuss the classifier. Section 6 presents the overall conclusions, whereas Section 5 describes the investigation and its findings.

## 2 Proposed methodology

**Fig. 1:**
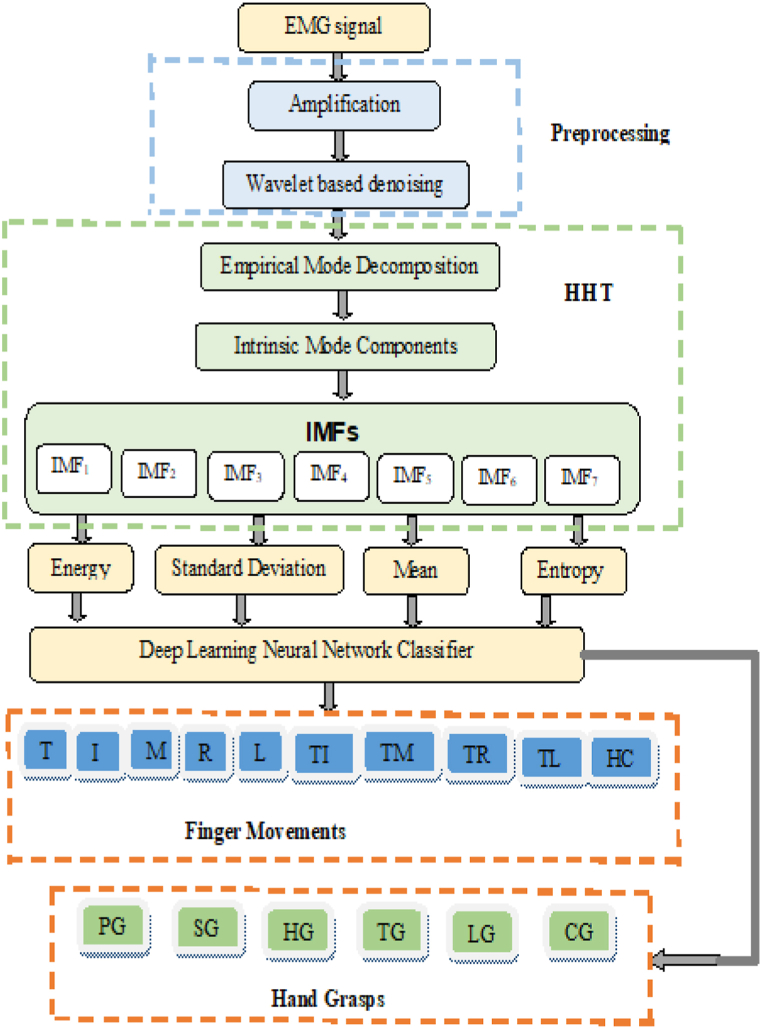
Diagrammatic illustration of the suggested work.

There are three main parts to the proposed method for classifying hand gestures: capturing and preprocessing signals, extracting features using EMD, and classification. The anticipated technique is revealed schematically in Fig. 1. The signal acquisition and preprocessing module eliminates unwanted noise by measuring the raw EMG signals and by means of appropriate denoising methods. They are amplified to improve the signal quality. It was with non-stationary signals that this method of adaptive data processing was born. Careful selection of the mother wavelet is required for the qualities that depend on the wavelet transform. A mother-wavelet-free HHT has recently been developed, which eliminates this issue. Decomposing the signal into its individual IMFs is the first step in implementing HHT using the EMD approach. It is common practice to provide the DNN with data for further classification after extracting relevant properties from the IMFs.

The accompanying parts provide an explanation of the suggested technique, which includes a full description of the framework.

### 2.1 Signal acquisition

**Fig. 2:**
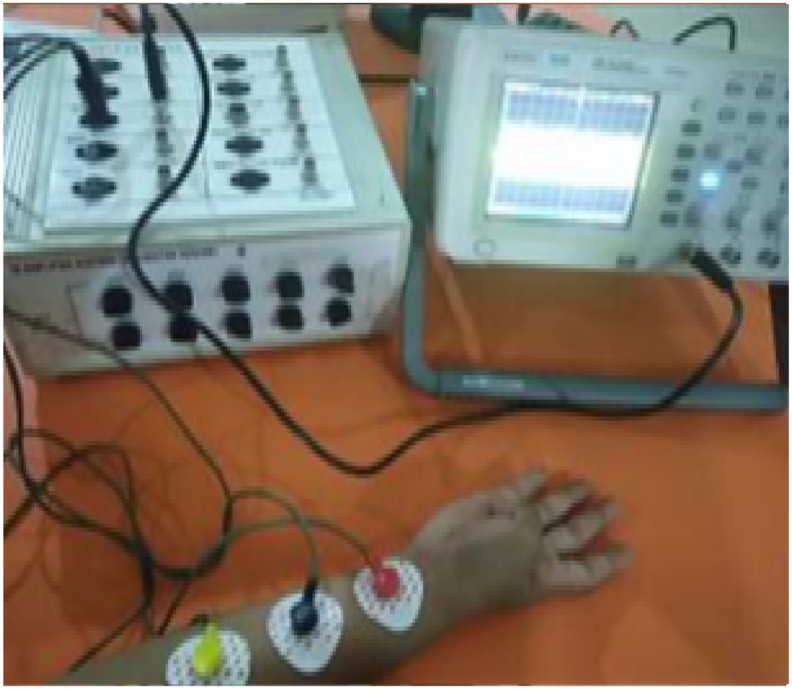
EMG dataset creation.

**Fig. 3:**
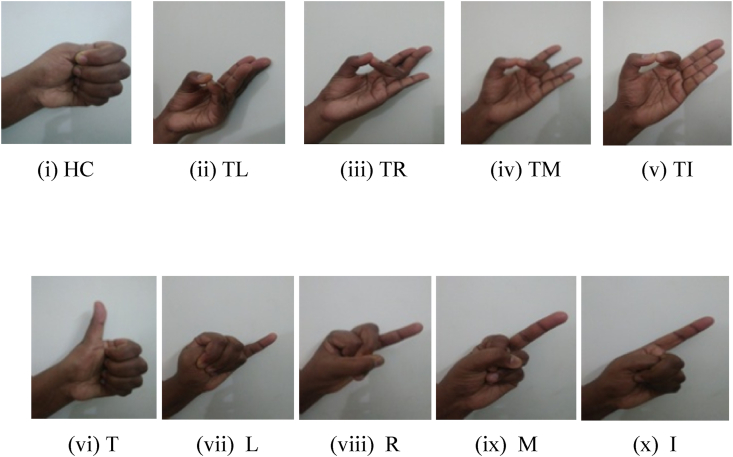
Finger movements.

There was not a single participant with a history of neurological or muscle disorders among the 27 people who took part, whose ages varied from 19 to 22. The gender breakdown was 15 women and 12 males. The arm was supported as exposed in Fig. 2. We used 70 % alcohol wipes for skin preparation and medical-grade epoxy resin tape to install the instruments. The two electrodes are 2 cm apart. Each subject had an electrically conducting reference electrode (Dermatrode Reference Electrode) attached to their wrist. After 5 s of doing a predetermined finger motion in a predetermined position, the participants were given a 5-s respite. Each action was carried out six times. Each experiment lasts 30 min, for a grand total of 180 min of data collection throughout all trials. Training and testing sets were constructed using the acquired data. In all, six trials were conducted, with two serving as training and four as testing [32]. In Fig. 3(i–x), we can see 10 distinct finger actions, all of which include bending the finger instances, such as HC, T-L,T-R, T-M, T-I,T, L, R, M, and I. The 'FM-dataset' is a collection of EMG signals recorded during finger movements.

**Fig. 4:**
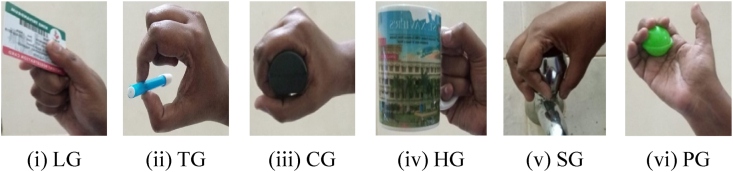
Hand grips.

The HG-dataset illustrates six various hand grasps that are used by humans. These hand grasps are as follows: the Lateral Grasp (LG), the Tip Grasp (TG), the Cylindrical Grasp (CG), the Hook Grasp (HG), the Spherical Grasp (SG), and the Palmar Grasp (PG) shown in Fig. 4(i-vi). For the purpose of acquiring all of the EMG signals, a sample rate of 500 Hz was used.

### 2.2 Amplification

This step, which is considered to be among the most significant procedures, is a part of the preparation stage. The amplitude of an EMG signal is rather modest, often lying within the range of tens to thousands of volts (μV). The signal must be amplified in order to get a greater gain, since this is the only way to achieve the desired result. The signal may also be contaminated by noise, which is still another option.

### 2.3 Denoising

The constant electromagnetic radiation emitted by all living things makes it almost impossible to imagine a world without some kind of background noise. Power plant emissions of 60 Hz (or 50 Hz) radiation are the primary cause of ambient noise complaints. The use of suitable filtering methods allows for the elimination of signal noise [33]. This approach uses wavelet-based denoising techniques to separate the important signal from the background noise.

**Fig. 5:**
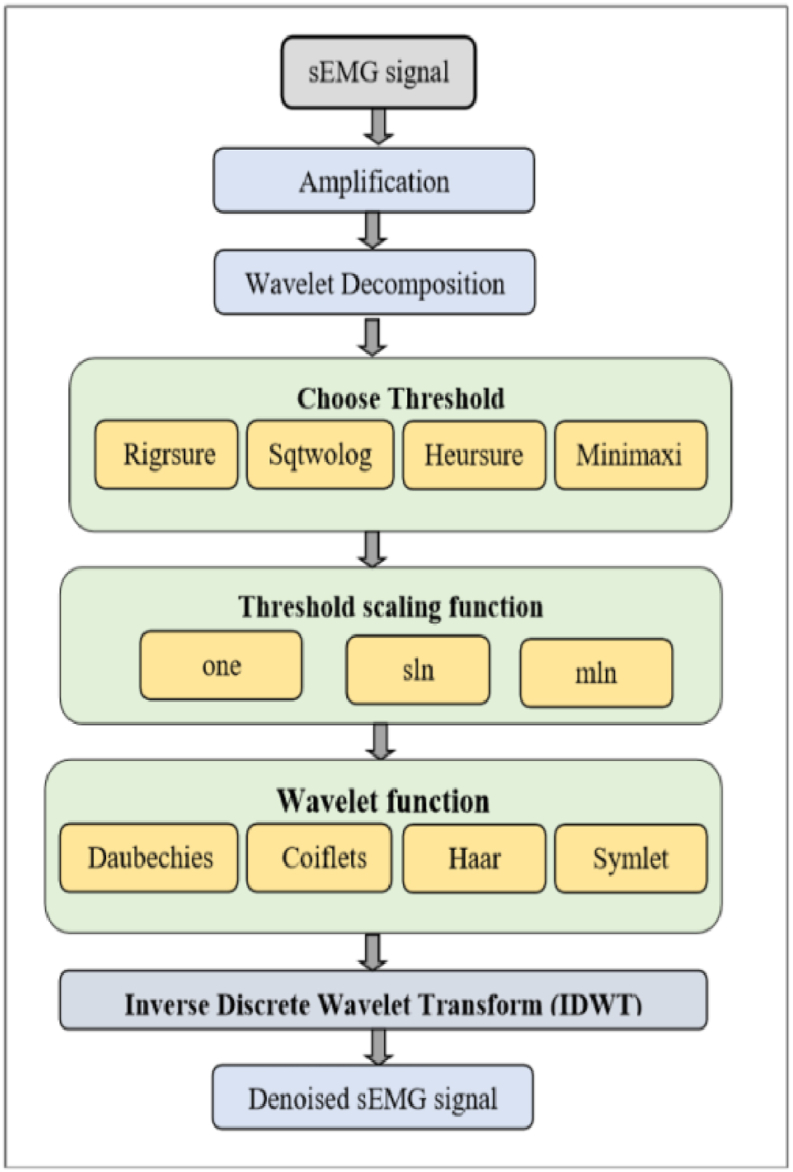
Wavelet-based denoising.

We may scale the signal's attributes using the wavelets. Consequently, they are able to remove the noise while extracting the signal's fundamental properties [34]. Because the Wavelet Transform sparsely captures signals, with most of the signal's features concentrated in a small number of large-magnitude wavelet coefficients, wavelet denoising is based on this principle which is shown in Fig. 5.

Due to the fact that they are considered noise, it is possible to eliminate unnecessary wavelet coefficients, which are defined as those that have no practical use, without causing a degradation in the signal quality. After obtaining the denoised signal via the use of the threshold, it is really possible to reconstruct the signal through the utilisation of the inverse wavelet transform.

## 3 HHT

For non-stationary and transient signals, the HHT outperforms conventional transform algorithms in terms of frequency resolution and time [35]. HHT employs EMD to simplify the complex signals by converting them into IMFs, or intermediate modulated signals.

### 3.1 Decomposition

Use of a method known as "Empirical Mode Decomposition" allows for the simplification of a signal obsessed by its constituent "Intrinsic Mode Functions" (IMFs). It serves as the foundation for the "Hilbert Huang Transform (HHT)". This research uses EMD to decompose the wavelet-based denoised signal and get IMFs.

It is possible to calculate the upper and lower envelopes of the signal by using the formulas that are shown below (1,2).

(1) $$U_{E}=e_{\max}\left\{X_{lk}\left(t\right)\right\}$$

(2) $$L_{E}=e_{\min}\left\{X_{lk}\left(t\right)\right\}$$

**Fig. 6:**
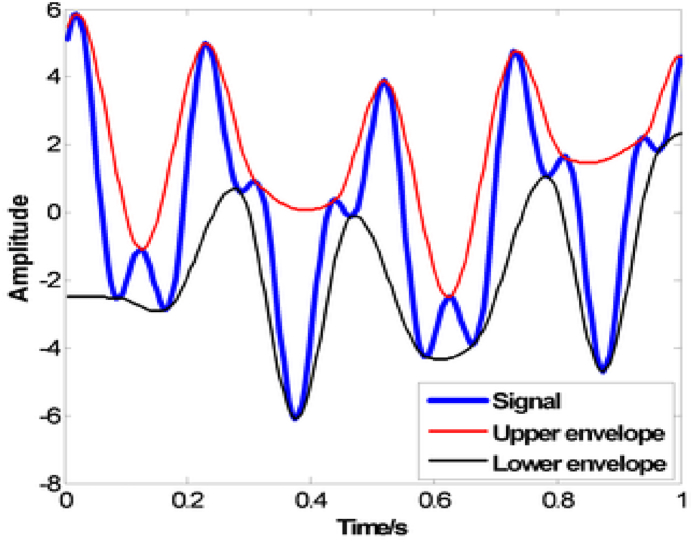
The signal of envelopes.

In this context, the maximum and minimum amplitudes of the input denoised signal X_lk_(t) are represented by the symbols e_max_ and e_min_, respectively. The lower envelope is shown by the symbol L_E_, while the upper envelope is represented by the symbol U_E_. The bottom and upper envelopes of a signal are shown in Fig. 6, which is a visual representation of the signal.

In order to determine the IMFs for the given signal, an iterative calculation is performed using a shifting method, which is abridgedunderneath.

**Table:** 

EMD Algorithm
Input: Denoised data Output:Denoised data IMFs Begin Step 1:Initialize the input's local maxima and minima values and the number of IMFs to be formed. Step 2: The envelopes are calculated using interpolation method. Step 3: Use Eqn. (5) to calculate the Mean. Step 4: Use Eqn. (6) to decompose the input. Step 5: If step 4 is invalid, the IMFs should becreated by shifting. Step 5.1: Extract the residue from the input. Step 5.2: $h_{i}\left(t\right)$ is calculated. Step 5.3: Use Eqn. (7) to calculate the Standard deviation. Step 5.4: If $r_{i}\left(t\right)\leq N$, check if $\sigma_{i}<\delta$ Step 5.5: do $\text{IM}F_{i}\left(t\right)\leftarrow h_{i}\left(t\right)$ Step 5.6: else do $h_{i}\left(t\right)\leftarrow h_{i+1}\left(t\right)$, $r_{i}\left(t\right)\leftarrow r_{i+1}\left(t\right)$ Step 5.7: Steps 5.1 to 5.6 are repeated Step 6:Steps 2 to 4 are repeated, until the anticipated number of IMFs is produced. End

Using Eqn. (3) and Eqn. (4), we can determine the mean of the denoised input $\mu \left\{X_{lk}\left(t\right)\right\}$ as:

(3) $$\mu \left\{X_{lk}\left(t\right)\right\}=\frac{U_{E}+L_{E}}{2}$$

The decomposed output d(t) is obtained by subtracting the input from the mean computed using Eqn. (4) [5].

(4) $$d\left(t\right)=X_{lk}\left(t\right)-\mu \left\{X_{lk}\left(t\right)\right\}$$

The first IMFs are generated by the shifting method. Finding the threshold value δ is the first step. Usually, δ falls around between 2 % and 3 % [36]. To get the standard deviation $\sigma_{i}\left(t\right)$ of the given data, you may perform the following equations (5), (6) [6]:

(5) $$\sigma_{i}\left(t\right)=\frac{\sum_{i=1}^{k}{\left|h_{i-1}\left(t\right)-h_{i}\left(t\right)\right|}^{2}}{h_{i-1}{\left(t\right)}^{2}}$$

where $h_{i}\left(t\right)\leftarrow r_{i}\left(t\right)$.

where $r_{i}\left(t\right)$ is the residual data from the input. In order to get genuine IMFs, the shifting process is iterated until the following halting requirements are satisfied.

1) There can be no more than a one-to-one difference between the total zero crossings and extrema in the data collection.
2) The maximum and minimum values for any given location must be zero and one, respectively, to establish the upper and lower envelops.

Finally, the signal that has been decomposed may be articulated.

(6) $$d\left\{X_{lk}\left(t\right)\right\}=\sum_{i=1}^{k}\sum_{j=1}^{l}IMF_{ij}\left(t\right)+r_{ij}\left(t\right)$$

This results in the creation of N number of IMFs that are capable of being processed singly. It is possible to write the IMFs that were mined from the signal in the form of an arrangement as follows equation (7):

(7) $$IMF\left(X_{lk}\right)=\left[\begin{array}{l} IMF_{1,1},IMF_{2,1},....IMF_{l1}\\ IMF_{1.2},IMF_{2,2},....IMF_{l2}\\ :\\ IMF_{1k},IMF_{2,k},....IMF_{lk} \end{array}\right]$$

### 3.2 EMD based feature extraction

The produced IMFs provide the basis for a variety of statistical properties, such as M, S, E, and U. The classifier may utilise the feature vector that is generated from these retrieved attributes to train itself for future classification tasks. The mathematical equations used to calculate these properties may be seen below, starting with equation (8) and continuing through equation (11).

1) Mean:

(8) $$M=\frac{1}{N}\sum_{i=1}^{N}X_{i}^{2}$$

2) Standard Deviation:

(9) $$STD=\sqrt{\frac{1}{N-1}\sum_{i=1}^{N}X_{i}^{2}}$$

3) Energy:

(10) $$E=\sqrt{\sum_{i=1}^{N}X_{i}^{2}}$$

4) Entropy:

(11) $$U=\sum_{i=1}^{N}X_{i}^{2}-\ln\;{\left(X_{i}\right)}^{2}$$

IMFs, which contain the most relevant information about the signal, are used to extract these statistical aspects from the signal.

## 4 DNN classification

**Fig. 7:**
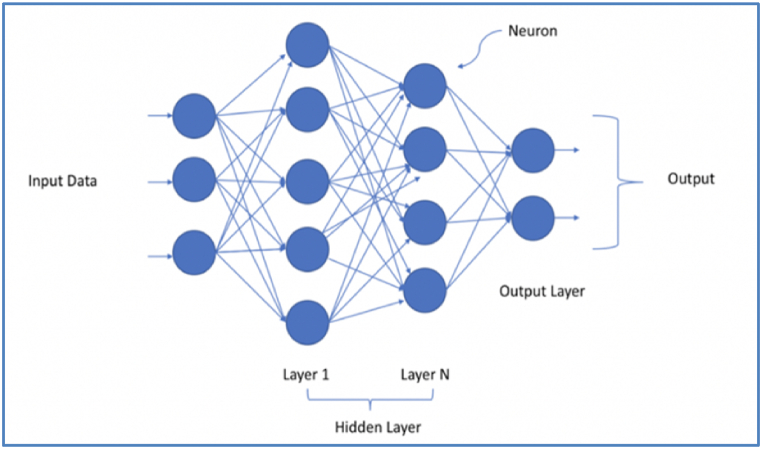
DNN's architecture.

A series of hidden layers are used to transfer the input from the input to the output layer [37]. The DNN's architecture is shown in Fig. 7.

**Fig. 8:**
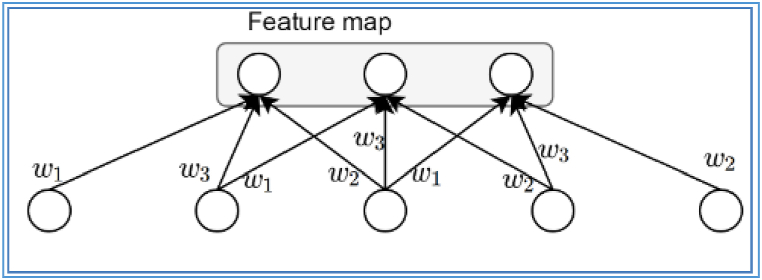
ANNweights.

A variety of DNNs are available. The ideal architecture to use for recognition and classification tasks is a CNN. The CNN recompenses a 'convolution' process, which is an essential that characterises the extent to which two functions overlap. Artificial neural networks (ANNs) use the convolution operation instead of matrix multiplication in any of the layers [38]. Convolution has as its principal objective the extraction of valuable characteristics from input data. The CNNs evaluate input by use of a grid-like structure and a number of "windows" referred to as filters [39]. By making use of a feature map that incorporates all input filters and their associated weights and biases, learning efficiency is enhanced [40]. With identical weights (w_1_, w_2_, w_3_), the three hidden units in Fig. 8 form a single feature map. Networks are trained using the back propagation method and their weights are rationalized by means of the gradient decent approach [41].

The DNN training algorithm may be summarised as follows.

Step 1 Initialization of the weights of the network is done in a random fashion.

Step 2 An method that is based on gradient descent and back propagation is used for the purpose of training the network.

Step 3 One takes a sample of the labelled data known as the batch.

Step 5 The error signal is engendered by the disparity among the values that were anticipated and those that were targeted.

Step 6 In order to get more accurate forecasts and to ensure that the weights are kept up to date.

Step 7 A portion of the gradient is subtracted from the cost function.

When a DNN is being trained, two different activation functions are looked at. In this example, the softmax activation unit and the corrected linear activation unit are both present and functioning. Mapping an output to a collection of inputs may be accomplished via the use of a rectified linear activation function. Utilising them gives the design of the network a non-linear quality that is a distinct advantage. It is possible to see the linear activation function in equation (12).

(12) $$f\left(x\right)=\left\{ \begin{array}{c} x,x>0\\ 0.01x,\text{otherwise} \end{array}\right.$$

Where x is the signal that is being input. This is accomplished by using the Softmax function in order to determine the probability distribution for the k output classes. The softmax activation function is shown in equation (13), which may be found here.

(13) $${\;p}_{j}=\frac{e^{x_{j}}}{\sum_{1}^{k}e^{x_{k}}}\;\text{for}\;j=1,\ldots k$$

The values of *p* that are output range from 0 to 1, and their total is 1.

## 5 Results and discussion

**Fig. 9:**
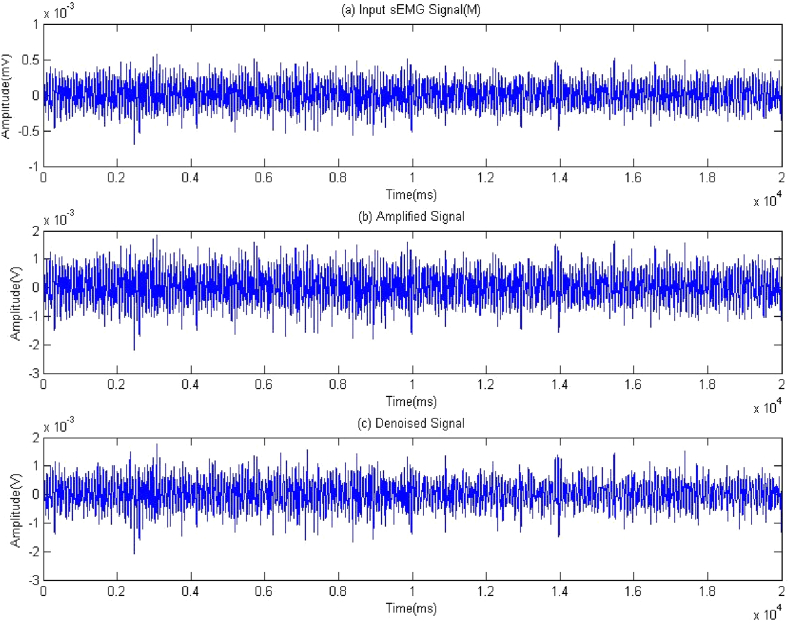
EMG signal along with its amplification.

Both the FM-dataset and the HG-dataset are samples of EMG signals that have been gathered. Similar to the previous datasets, these datasets are produced by following the technique described in section 2.1. By introducing noise to an EMG signal, it is possible to lower the signal's quality. For this reason, it is essential to ensure that the input signals are improved and cleaned up. The EMG signal related to the movement of the middle finger is seen in Fig. 9, together with its amplified and denoised equivalents.

**Fig. 10:**
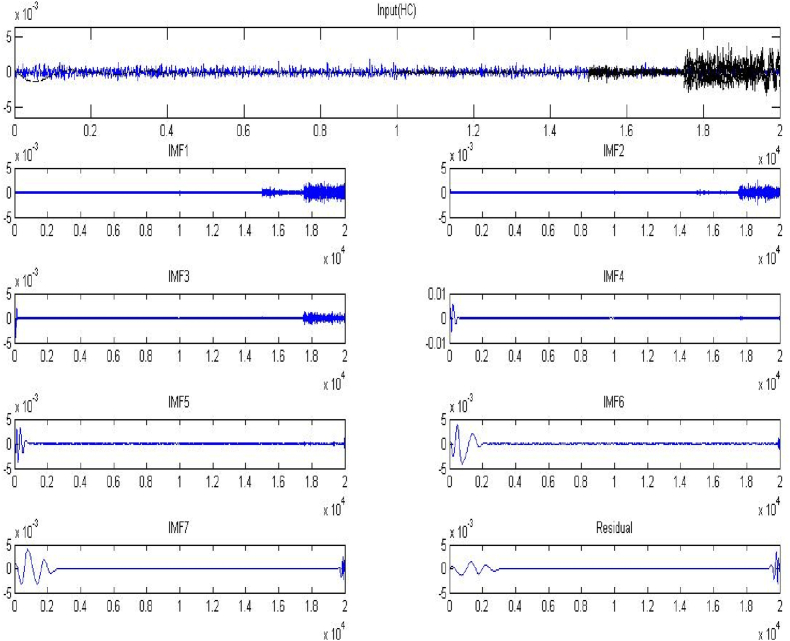
EMG signal for Hand close andIMFs resulting.

**Fig. 11:**
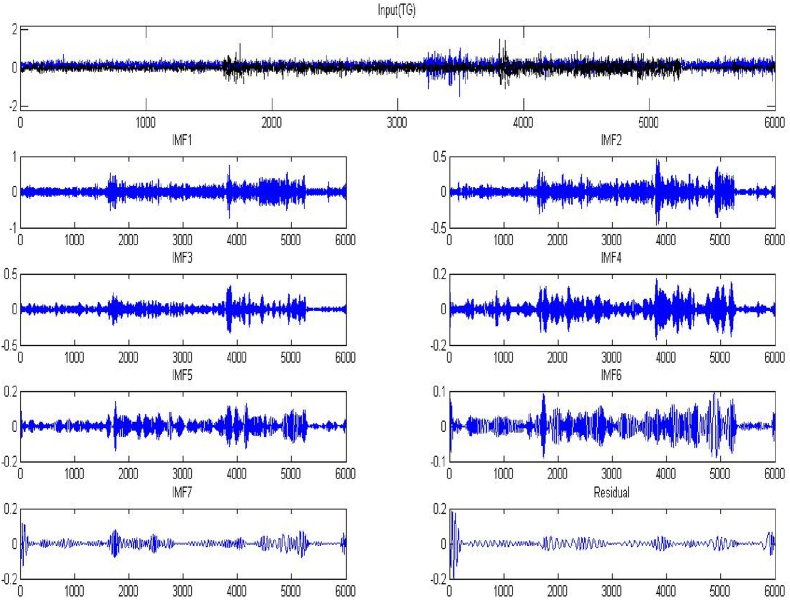
EMG signal for Tip Grasp and IMFs resulting.

Using the EMD method, we are able to separate the individual IMFs from the EMG data that has already been processed. The first base component has the widest frequency band among the decomposed portions, whereas the final decomposed element has the smallest. The EMG signals for the HC and TG are shown in Fig. 10, Fig. 11, respectively, along with the resulting IMFs.

**Fig. 12:**
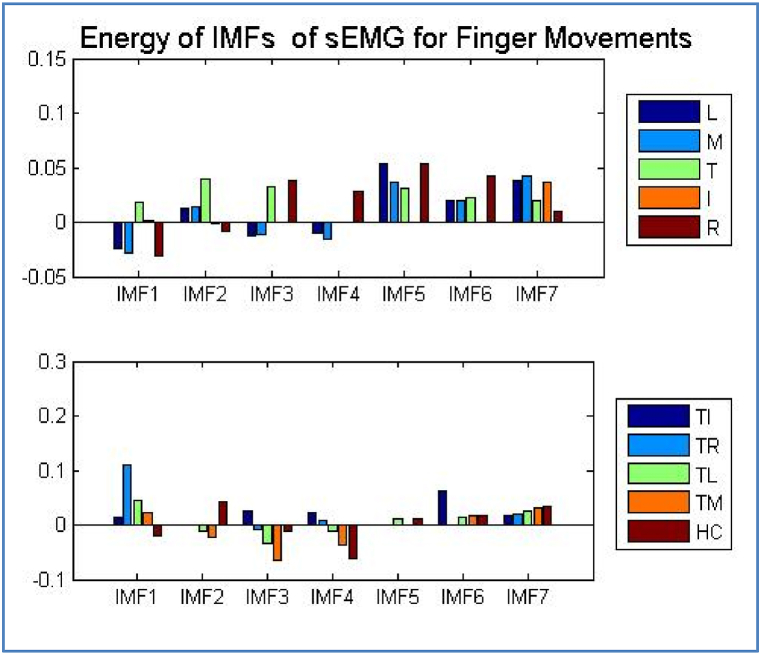
Energy values of finger actions.

Mean, Energy, Entropy, and Standard Deviation are some of the statistical metrics that may be extracted from the decomposed IMFs using Equations (8), (9), (10), (11)). Fig. 12 demonstrates the magnitudes of the EMG IMFs in relation to the finger actions. It is evident that the Energy levels vary throughout all IMFs.

**Fig. 13:**
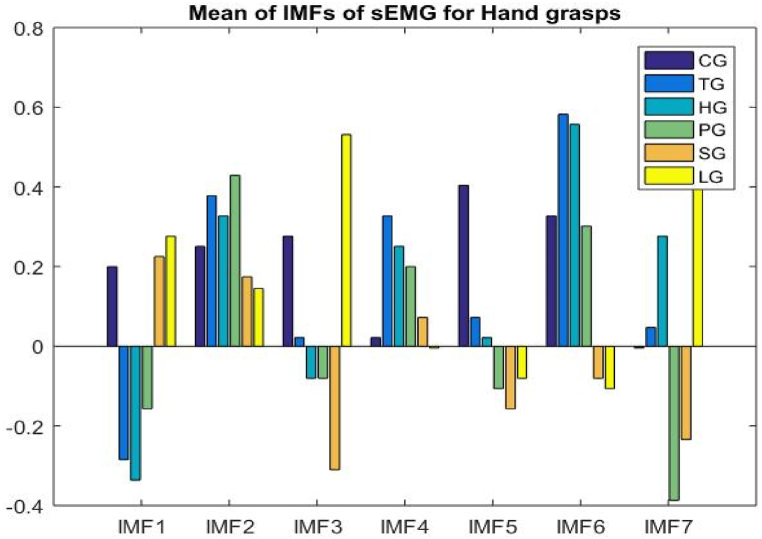
IMFs of EMGMean values for the hand grasps.

A representation of the average of the IMFs obtained from the electromyography of the hand grasps may be seen in Fig. 13.

**Fig. 14:**
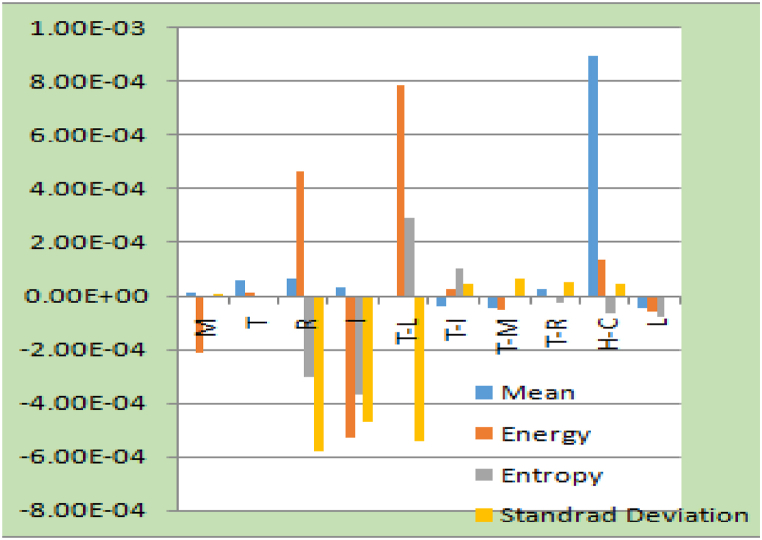
Feature extraction of IMF1.

**Fig. 15:**
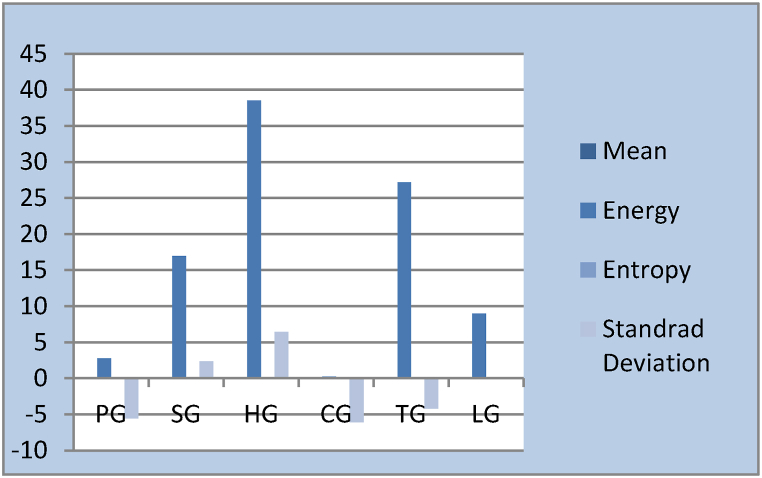
Feature extraction of IMF1.

Feature extractions from IMF1 are shown in Fig. 14, Fig. 15, respectively, for each of the datasets.

Fig. 14 shows that the various finger movements have their own unique properties. In Fig. 15, we can see how IMF1 stacks up against different hand grasps and how these features change over different hand grips.

In Table 1, Table 2, we can see the percentage of accuracy in classification achieved using the DNN classifier for both datasets. Properly categorised values are shown by the diagonal parts of Table 1, Table 2, whereas misclassified values are shown by the off-diagonal sections.

**Table 1:** FM-dataset.

Finger Actions	H–C	T-L	T-R	T-M	T-I	T	L	R	M	I
**H–C**	0.0	0.0	1.0	0.0	1.0	0.0	0.0	0.0	0.0	98
**T-L**	0.0	0.0	0.0	0.0	0.0	0.0	0.0	1.0	99	0.0
**T-R**	0.0	0.0	1.0	0.0	0.0	0.0	0.0	99	1.0	0.0
**T-M**	0.0	0.0	0.0	0.0	0.0	0.0	98	0.0	1.0	1.0
**T-I**	0.0	0.0	0.0	0.0	2.0	97	0.0	0.0	0.0	1.0
**T**	0.0	0.0	1.0	1.0	99	0.0	0.0	1.0	0.0	0.0
**L**	0.0	0.0	0.0	99	0.0	0.0	1.0	0.0	0.0	0.0
**R**	0.0	0.0	98	1.0	0.0	0.0	0.0	1.0	0.0	0.0
**M**	0.0	99	0.0	0.0	0.0	0.0	1.0	0.0	0.0	0.0
**I**	98	0.0	0.0	1.0	0.0	0.0	0.0	1.0	0.0	0.0

**Table 2:** Accurate classification for HG-dataset.

	LG	TG	CG	HG	SG	PG
**LG**	1.0	0.0	0.0	0.0	0.0	98
**TG**	0.0	0.0	0.0	1.0	99	0.0
**CG**	0.0	0.0	0.0	99	1.0	0.0
**HG**	0.0	0.0	98	0.0	1.0	1.0
**SG**	2.0	97	0.0	0.0	0.0	1.0
**PG**	99	0.0	0.0	1.0	0.0	0.0

The accuracy, recall, and discrimination of each classifier are evaluated as follows equation (14), (15), (16):

(14) $$Accuracy=\frac{TP+TN}{TP+TN+FP+FN}$$

(15) $$Sensitivity=\frac{TP}{TP+FN}$$

(16) $$Specificity=\frac{TN}{TN+FP}$$

Where,*TN* stands for True Negative, *TP* for True Positive, *FN* for False Negative, and *FP* for False Positive, all of which have been rightly rejected.

According to the data shown in Table 1, Table 2, the overall accuracy of all of the individual finger motions is more than 97 % respectively. An average classification rate of 98.5 % was achieved as a consequence of this process. Furthermore, the HG-dataset offers a 98.7 percent classification rate. Table 3 presents a comparison of the findings obtained for the both the collected and open EMG Ninapro datasets. This comparison is made with regard to the specificity and sensitivity of the DNN classifier.

**Table 3:** DNN classifier performance.

Dataset	Specificity	Sensitivity	Accuracy
**FM-Ninapro dataset**	91.70	90.40	98.70
**FM-dataset**	98.50	90.10	91.40
**HG-Ninapro dataset**	90.40	91.30	98.60
**HG-dataset**	98.70	91.20	90.60

Table 4 displays the results, which compare the proposed method's classification accuracy to other published methodologies.

**Table 4:** Comparing the suggested approach to alternative approaches.

features	Classifier Type	Classification Rate	
		FM-data	HG- data
**Time domain features**	FFNN	75.30	75.00
	CFNN	75.50	75.40
	KNN	75.90	75.40
	SVM	76.30	76.00
	DNN	78.50	78.70
**Frequency domain features**	FFNN	82.30	83.00
	CFNN	83.50	82.40
	KNN	83.90	83.40
	SVM	84.30	84.00
	DNN	84.50	84.70
**DWT**	FFNN	85.30	85.00
	CFNN	85.50	85.40
	KNN	85.90	85.40
	SVM	86.30	86.00
	DNN	88.50	88.70
**WPT**	FFNN	92.30	93.00
	CFNN	93.50	92.40
	KNN	93.90	93.40
	SVM	94.30	94.00
	DNN	94.50	94.70
**HHT**	FFNN	**95.30**	**95.00**
	CFNN	**95.50**	**95.40**
	KNN	**95.90**	**95.40**
	SVM	**97.30**	**97.10**
	DNN	**99.60**	**99.20**

Table 4 demonstrates that out of all the classifiers tested, the HHT-based feature extraction approach with the DNN classifier produced the best results. Time, frequency, and wavelet are the three domains that are taken into account while conducting comparisons. During the classification phase, many methodologies are taken into consideration, such as KNN, FFNN, CFNN and SVM.

### 5.1 Novelty of the proposed framework

The classification rate is low due to the fact that the features recovered from raw EMG signals do not include any information that is contained in the frequency content of the signal.

On the other hand, frequency domain feature sets alone enhance classification accuracy. It can be enhanced by means of DWT and WPT, which look at the signal in the frequency and time domains, respectively. Using feature sets based on WPT results in a classification rate ranging from 90 % to 94 %, as seen in the table above. While DWT's accuracy enhancement relies on the mother wavelet being correctly selected, this is also one of its main drawbacks. Finally, as HHT is not dependent on a mother wavelet, increasing the classification rate using HHT-based feature sets eliminates the need for additional methods to detect mother wavelets.

Classifiers play a pivotal role in improving performance and increasing the classification rate. Recognitions to its multi-hidden-layer architecture, the proposed DNN classifier significantly improve the classification rate. One novel aspect of the proposed system is the DNN classifier; another is the HHT-based feature set.

## 6 Conclusion

In this study, an efficient approach for classifying hand gestures has been suggested. The HHT and DNN are the two methods that have been proposed.

• In order to get the most critical qualities of the EMG signal, the required topographies are haul out from its IMFs. We have shown the findings of the categorization of hand gestures and finger motions.
• The DNN classifier with improved properties outperforms the others.
• When compared to the other approaches, the experimental findings suggest that the combined use of HHT and DNN provided better outcomes.

Additional signal processing methods, such as frequency spectrum estimation, for feature extraction, may be introduced to expand the current work. The suggested signal processing methods might be executed on VLSI processors for use in real-time applications. It is also possible to use ANN architectures such as ART and SVM for the purpose of automated hand gesture identification.

## Funding

The authors extend their appreciation to the Researchers Supporting Project number (RSPD2024R698), 10.13039/501100002383King Saud University, Riyadh, Saudi Arabia for funding this research work.

## Ethical statement

The authors jointly declare that the consent has been obtained and all the participants are aware of intended publication.

## Data availability

The datasets used and/or analysed during the current study are available from the corresponding author on request.

## CRediT authorship contribution statement

**Mary Vasanthi S:** Writing – original draft, Conceptualization. **Haiter Lenin A:** Writing – review & editing, Data curation. **Yasser Fouad:** Validation, Funding acquisition. **Manzoore Elahi M. Soudagar:** Writing – review & editing, Project administration, Software.

## Declaration of competing interest

The authors declare that they have no known competing financial interests or personal relationships that could have appeared to influence the work reported in this paper.
